# A review of critical perspectives on private land conservation in academic literature

**DOI:** 10.1007/s13280-019-01258-y

**Published:** 2019-10-12

**Authors:** Jennifer Gooden, Michael ‘t Sas-Rolfes

**Affiliations:** Oxford University Centre for the Environment, Smith School of Enterprise and the Environment, Oxford University School of Geography and the Environment, South Parks Road, Oxford, OX1 3QY UK

**Keywords:** Conservation effectiveness, Governance, Neocolonialism, Neoliberalism, Private land conservation, Privately protected area

## Abstract

In recent years, private land conservation has increased in profile among policymakers and academics. Conservation initiatives on privately owned land help to mitigate global biodiversity loss and introduce new actors to conservation. However, they have also been the subject of numerous critical accounts. This review catalogs issues that emerge in critical literature, identifying 25 themes, classified into three groups: Implementation Effectiveness, Value Conflict, and Economic Inefficiency. Gaps in the literature include the need for broader geographic coverage; assessment of the issues’ specificity to private land conservation; and evaluation of the extent to which issues in the literature reflect broader societal values. The literature’s strong emphasis on value conflict suggests that greater attention to governance effectiveness may steer private land conservation toward practices that are more just, equitable, and representative and lead to increased societal support. We recommend further research to address identified gaps, with a greater orientation toward inclusive governance.

## Introduction

Since the creation of the first national parks in the late eighteenth century, states have been the primary agents of land conservation (Adams 2004). However, for various reasons, it is increasingly apparent that government action alone will be insufficient to reach global protected area targets (Watson et al. 2014; Butchart et al. 2015) and that private entities have a significant role to play.

A 2014 report on privately protected areas by the World Commission on Protected Areas (Stolton et al. 2014) raised the international profile of private land conservation (PLC) among policymakers, nongovernmental organizations (NGOs), and academics. Several governments have recently established PLC mechanisms. For example, in 2016, the government of Chile passed legislation to permit the *derecho real*, a newly codified conservation property right (ILCN 2016). In Catalonia, a land stewardship network called Xarxa de Custòdia del Territori has made progress to secure legislation enabling land stewardship agreements and tax incentives (Brandehof 2018). NGOs and networks have also been established specifically to promote PLC; prominent examples include the International Land Conservation Network (formed in 2014), the European Land Conservation Network (2017), the Australian Land Conservation Alliance (2011), and Así Conserva Chile (2010). Academic research on PLC is also increasing, with a Scopus search showing a more than tenfold increase in the number of articles published annually from 2007 to 2018.

There are many reasons for conservationists to support increased attention to PLC (Gooden 2019a, b). Land use change is a threat to biodiversity both in and out of protected areas, and any increase in efforts to protect land of high conservation value, regardless of ownership, could help stem the loss of biodiversity (Tilman et al. 2017). While privately protected areas are generally smaller than state-protected areas (Stolton et al. 2014), this is not always the case, and they can serve important functions as corridors and buffer zones for larger protected areas (Willis et al. 2012). Not the least, the introduction of new actors into conservation, particularly those with experience outside the fields of conservation NGOs, government agencies, and academic research, may increase potential for innovation and entrepreneurship (Moon et al. 2014).

Despite this positive potential of PLC, many academics view it as problematic. The rise of PLC in policy and practice has spawned a body of critical work, grounded in diverse epistemological and methodological approaches. For example, scholars have highlighted that international land acquisition for conservation, especially in developing countries, risks importing values associated with neocolonialism, elitism, and land grabbing (Ramutsindela et al. 2011). Others see PLC as a product of neoliberal roll-back of the state (Holmes 2011) or a less than optimally effective conservation mechanism (Clements and Cumming 2017). These various criticisms arise from diverse disciplines and are published across a broad range of academic journals. To our knowledge, they have not been cataloged in any structured way; this is a gap we seek to address in this review.

This exercise has several benefits. Drawing attention to areas where PLC is purportedly not performing well can catalyze improvement of practices, potentially leading to conservation mechanisms that work more effectively or more compatibly with other societal goals. Dissenting voices help situate conservation in the context of other social values (e.g., equity, local self-determination), which vary across space and time (Knox-Hayes 2015) and with which some conservation visions may conflict (e.g., Yung et al. 2003). Critical perspectives can improve the quality of group decisions, in part by avoiding “groupthink” (Postmes et al. 2001; Reed 2008). Because some of the critical literature is published in journals not widely read by conservation scientists and practitioners, this review also aims to facilitate engagement with diverse perspectives and to promote interdisciplinary thinking, which is increasingly acknowledged as essential for effective collective approaches to tackling complex environmental problems (Lélé and Norgaard 2005).

There are currently at least 50 definitions related to privately protected areas in use (Stolton et al. 2014). For the purposes of this review, we define PLC broadly, as any efforts by private actors to conserve terrestrial biodiversity on land they own or control. Private actors include all entities not classed under the International Union for the Conservation of Nature (IUCN) definitions for public/state governance or indigenous and community governance, including individuals and groups of individuals, NGOs, corporations, for-profit owners, research institutions, and religious institutions (Stolton et al. 2014). The IUCN has also specifically defined privately protected areas (PPAs) as protected areas under private governance, with a protected area being “an area of land and/or sea especially dedicated to the protection and maintenance of biological diversity, and of natural and associated cultural resources, and managed through legal or other effective means” (Stolton et al. 2008). However, in this paper, we concern ourselves less with whether the included papers adhere to the specific definition of a PPA and more with the exercise of cataloging how and why PLC is an object of criticism.

## Methods

Our objective for this review was to catalog the various ways in which PLC is criticized in the academic literature. We used an aggregative approach to conduct a ‘stocktaking’ activity, adopting Booth et al.’s (2016) SALSA method, which consists of four phases: Search, Appraisal, Synthesis, and Analysis. Although we were not evaluating evidence for a particular hypothesis, we documented each phase for transparency, auditability, and reproducibility.

In the Search phase, we searched Scopus and Web of Science in November 2017, using the terms listed in Table 1, which were derived from a sample of the known literature on the topic (Gooden 2018). This resulted in 954 unique documents. The Appraisal phase comprised an assessment of eligibility. We included only journal articles that met the criteria outlined in Box 1. For efficiency, we conducted the screening in two stages, removing irrelevant articles at each stage. The first screen, by title and abstract, narrowed the pool of articles to 151, and the second screen, by full-text, resulted in 57 articles for the review.

**Table 1:** Search terms used in Scopus and Web of Science to search for relevant documents

Search term	Items returned in Scopus	Items returned in Web of Science
Private land conservation	109	104
Private nature reserve	43	65
Private protected area	258	30
Privately protected area	28	0
Private wildlife ranch	28	0
Private wildlife ranches	18	2
Private reserve	270	186
Green grab	127	5
“Land grab” AND conservation	24	25

**Box 1:** Inclusion criteria for article screening

Concerned with land conservation, not species conservation (where distinct from land conservation, such as privately-owned wildlife rehabilitation centers)	
Focused on private land ownership, which includes individuals, groups, corporations, NGOs, research institutions, and religious organizations (Stolton et al. 2014)	
Related to governance, ethics, social issues, social impacts, politics, political economy	
Included ecotourism, private game reserves, conservation and development initiatives, and other mechanisms sometimes used to finance private land conservation	
Defined conservation in terms of biodiversity or ecosystem protection; did not include conservation exclusively focused on cultural, historical, or architectural elements	
Excluded analyses that focused on drivers of private land conservation, such as surveys of landowner attitudes, values, or preferences	

We applied a thematic approach to the Synthesis phase, generating the themes reported in the Results section. We read each of the articles, noting any points of criticism about PLC. We used an inductive process, open-coding these points using terms that reflected the nature of the critique, such as *neocolonialism* or *elitism*, using Willig’s (2013) approach to thematic coding. We recorded our findings in a database that included the themes, theoretical lens (if any), topic (e.g., covenants, private nature reserves), and geographic region for each article. Some concepts could be categorized in more than one way; we have reported the themes in detail so readers can view the constituent topics of each theme.

In many cases, the authors of the papers collected in this review attributed the critical points to others; in such situations, we have attributed the arguments to the sources in which they were encountered, rather than the original. Because the review aimed to capture the full range of critical themes, we included all themes, even if they appeared in only one article, and we included all themes encountered in each source article, regardless of whether they were reflective of the overall tone or argument of the author(s). We neither coded nor reported arguments in favor of PLC or rebuttals to criticism.

**Fig. 1:**
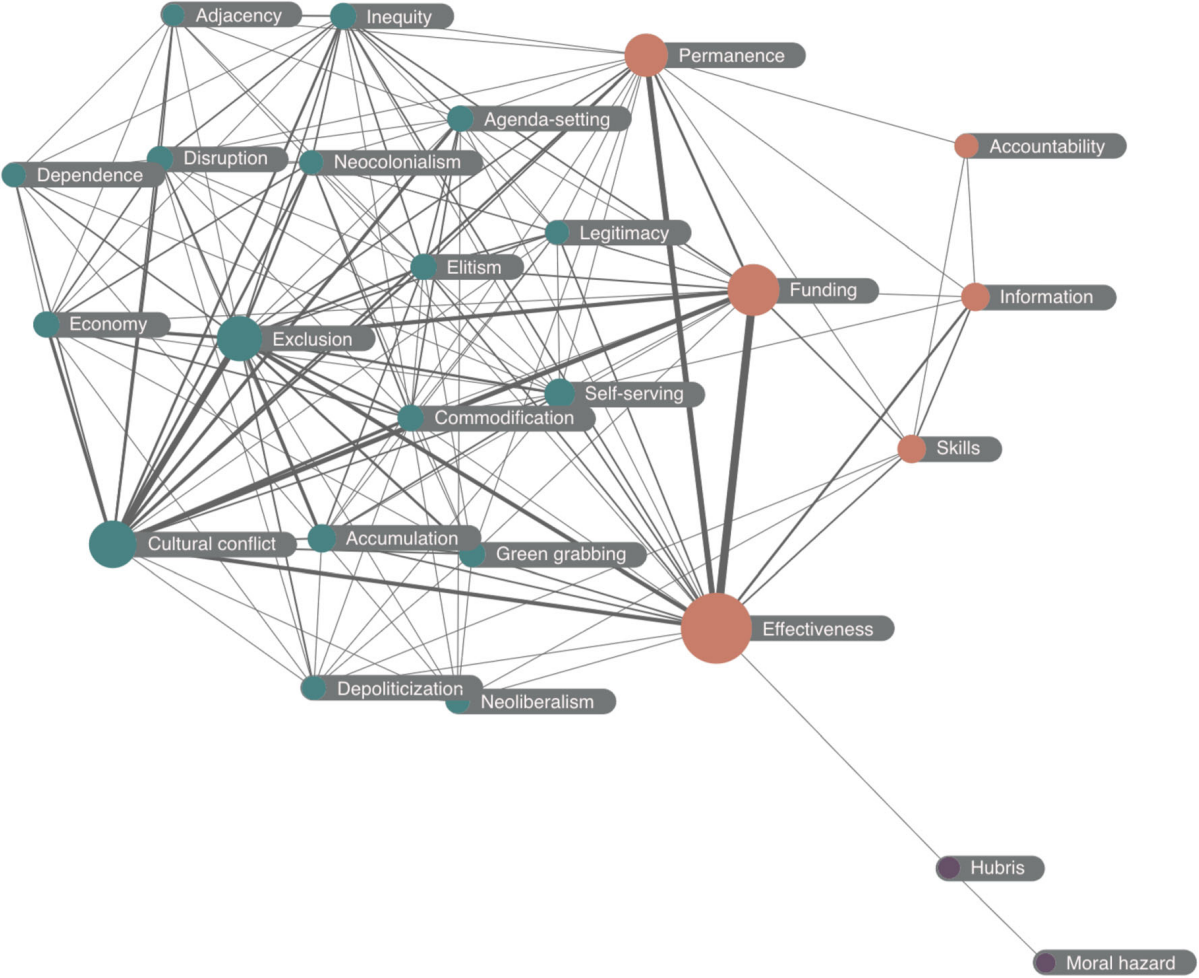
Network diagram illustrating connections among themes in 57 source articles. The size of the nodes (circles) represents the number of papers that include each theme, and the weight of the edges (connecting lines) represents the number of co-occurrences of each pair of themes within the articles. Peach: Group 1. Teal: Group 2. Plum: Group 3

In the Synthesis phase, we explored how the emergent critical themes were related to each other by visualizing the connections between themes (Fig. 1). We created two data tables: a node count, which listed each theme and the number of articles in which it appeared, and an edge count, which recorded the number of times every given pair of themes co-occurred in an article. These data were graphed using Polinode network analysis software (Pitts 2016) using a force-directed graphing algorithm. With the aid of the network diagram, we clustered the 25 themes into three overarching groups, which we describe in Results.

In the Analysis phase, reported in the Discussion section, we explored what we found and what was missing, drawing conclusions about the results of the synthesis and their generalizability to the context (Booth et al. 2016). We also considered our findings in relation to the existing frameworks in the literature to explore the meaning of our results to the study and practice of conservation.

## Results

The Search and Analysis phases identified 25 themes (Table 2), clustered into three groups: Group 1: Implementation Effectiveness, which includes concerns typically expressed by environmental scientists and practicing conservationists; Group 2: Value Conflict, which includes normative concerns expressed by scholars of political ecology; and Group 3: Economic Inefficiency, a topic of concern to neoclassical economists. As illustrated by the network diagram (Fig. 1), the themes are interrelated, and some overlap exists. In this section, we summarize the literature within each group and theme and conclude with some general observations.

**Table 2:** Number of source articles that contributed to each coded theme

Theme	Number of sources	Theme	Number of sources
Accountability	3	Exclusion	13
Accumulation	5	Funding	16
Adjacency	2	Green grabbing	4
Agenda-setting	4	Hubris	2
Neocolonialism	3	Inequity	4
Commodification	4	Information	5
Cultural conflict	14	Legitimacy	3
Dependence	3	Moral hazard	1
Depoliticization	3	Neoliberalism	3
Disruption	4	Permanence	12
Economy	4	Self-serving	6
Effectiveness	25	Skills	5
Elitism	4		

### Group 1: Implementation Effectiveness

Articles with themes in Group 1: Implementation Effectiveness tend to focus on practical issues of implementation, generally adopting the premise that conservation and effectiveness are universally understood and accepted. Most take PLC’s utility as a conservation mechanism as a given. To the extent they make comparisons with other land uses, they tend to benchmark against other protected areas (usually state protected areas), rather than competing land uses, such as agriculture or extractive industry.

#### Permanence

For various reasons, some landowners do not seek legal protection for conserved land. For example, some landowners may be deterred by the permanence of easements, particularly when there is financial risk involved (Freese et al. 2010). As a result, such lands may be vulnerable to ownership change or the whims of landowners (Leménager et al. 2014; Murray et al. 2015; Mitchell et al. 2017). Most conservationists, see permanent protection as the gold standard for land conservation, and one of the most prominent concerns about PLC is whether land can be permanently protected (Langholz and Krug 2004). Many authors report insecure and tenuous PLC efforts due to a lack of a national legal or regulatory structure for permanent protection (Langholz 2009; Pasquini et al. 2011; Kamal et al. 2015a; Bingham et al. 2017; Mitchell et al. 2017). Even where legal mechanisms are in place, they are not always permanent, as is the case for integrated conservation and development programs, which can be temporary solutions (Serenari et al. 2015).

In other cases, permanence concerns result from the difficulty of enforcing protection. For example, the technical documents that constitute easement agreements require a high level of legal sophistication. If the purpose of the easement is not specified in a way that is relevant to the future, the easement may become susceptible to legal challenges over time (Rissman et al. 2007). Land trust legal defense and enforcement challenges in the US are common, varied, expensive, and increasing, adding to the funding burden of the land trust movement (Rissman and Butsic 2011).

#### Effectiveness

Effectiveness is a large theme covering many elements, subject to varying interpretations among authors. Privately protected areas (PPAs) tend to be smaller than their state-owned counterparts, and thus associated with more fencing, roads, and conflicting adjacent land uses, leaving properties fragmented with ecological consequences (Freese et al. 2010; Kamal et al. 2015a). In addition to ecological challenges, the small scale of some properties can also lead to institutional challenges if management scales are too small for individual stakeholders to realize the full benefit and cost of conservation (Kreuter et al. 2010; Sundaresan and Riginos 2010). Furthermore, land ownership is not always accompanied by full use rights such that some factors are beyond the landowner’s control (Bingham et al. 2017).

Concerns about prioritization and decision-making include excessive focus on quantity over quality (Kamal et al. 2015b). Some conservation land trust organizations fail to include important factors in their acquisition decision-making, using individualistic and opportunistic parcel-level protection efforts rather than a systemic landscape-scale process (Ryan et al. 2014). Gaps include consideration of scale, parcel shape, leveraging, model iteration, and social and historical factors.

Monitoring effectiveness is an ongoing issue (de Santo 2012). A study of easements in the US (Rissman et al. 2007) found that only 20% had quantitative monitoring programs. Qualitative assessments suggested the status of conservation targets was steady or improving on nearly all easements, but the authors found this information subjective and potentially unreliable. The same study found that ecological monitoring was limited by funding, technical expertise, and staff time. Similarly, in Australia’s covenanting programs, organizations measured outcomes inconsistently, making it difficult for researchers to identify positive biodiversity outcomes across the covenanting programs in a systematic way (Fitzsimons and Carr 2014).

Some PLC programs may not adequately consider long-term ecological or social dynamics in their actions (Lindsay 2016). The Canadian Boreal Forest Agreement, for example, did not anticipate effects of climate change, which by 2080 may render areas unsuitable for caribou, the flagship species of the agreement (Murray et al. 2015). With the potential for historical ecological benchmarks to become an even more difficult restoration target into the future, Cooke and Corbo-Perkins (2018) argue there is a need to consider what conservationists aim to achieve through PLC restoration work, pointing to potential time scale mismatches between initiatives on individual land parcels and the need to engage with dynamic processes at the landscape level.

Several problems arise when PPAs must cover their costs by generating revenue through activities such as ecotourism or game ranching. PPAs that rely on ecotourism can risk degrading the resource they were set up to conserve (de Santo 2012). Even when ecotourism is carried out with a sincere interest in conservation, managers may lack a thorough understanding of ecosystem processes (Barany et al. 2001). Due to PPAs’ need for revenue, tourist preferences or other market drivers may affect decision-making (Cousins et al. 2008). Optimizing for nature-based tourism income may not equate to achieving optimal biodiversity outcomes (Baum et al. 2017). Practices of concern include overstocking wildlife to enhance opportunities for viewing and shooting (Clements et al. 2016; Clements and Cumming 2017); introducing extralimital species (Clements and Cumming 2017); undermining the genetic integrity of wildlife through selective breeding practices (Blackmore 2017); altering animals’ natural behavior through feeding, breeding, and other activities (Langholz 2010); maintaining animals in unnatural or abusive conditions (e.g., private zoos) (Langholz 2010); offering “canned hunting” opportunities that violate the principle of fair chase (Langholz 2010); persecuting predators to protect valuable game (Cousins et al. 2008); and neglecting less charismatic species that lack tourist appeal (Sundaresan and Riginos 2010).

Several have noted the vulnerabilities that result from PLC’s status as a voluntary activity. Because PLC depends on landowners being willing and aware of opportunities, important habitats may not be represented (Ladle et al. 2014; Ryan et al. 2014). In Poland, for example, Kamal et al. (2015b) report that landowners have little interest in conservation and that PLC may thus have limited feasibility. In Chile, most PPAs are concentrated in the south, so ecosystem coverage is unrepresentative (Tecklin and Sepulveda 2014). Likewise, the Canadian Boreal Forest Agreement, covering 4.6% of the boreal region of Canada, does not include most boreal ecozones (Murray et al. 2015). In addition, other threats such as water or mineral extraction may not be addressed through mechanisms like conservation easements if the surface landowner does not hold water rights or mineral, oil, and gas subsurface rights (Rissman et al. 2007). Consequently, PLC is only a partial solution to conservation needs, applicable to certain places with certain forms of biodiversity (Holmes 2015).

#### Funding and financial sustainability

Like protected areas in general, PPAs are vulnerable to funding problems. Private conservation landowners and reserve managers identify budget issues to be among their biggest concerns (Langholz 1996; Fitzsimons 2015), raising questions over whether planned conservation measures are feasible (Buergin 2016; Cooke and Corbo-Perkins 2018). Land trusts in the US cite a lack of funding among their top three challenges (Ryan et al. 2014).

In some cases, private entities acquire land without strategic financing procedures. This could include securing funding for property acquisition but not management, which adversely affects the long-term maintenance of properties (Pasquini et al. 2011; Farr et al. 2018). Overemphasis on start-up over management costs appears to be common (de Santo 2012). When conservation is dependent on income generated, PPAs are vulnerable to factors outside local control (Langholz and Krug 2004; Sundaresan and Riginos 2010; Baum et al. 2017). This can result in tradeoffs between short-term profit and long-term sustainability (Barany et al. 2001).

In other cases, even start-up costs for land acquisition or bureaucratic hurdles are beyond the reach of entities that wish to engage in PLC. Barany et al. (2001) detail the case of landowners in Nicaragua who were unable to conserve their land as they wished because they had no working capital and were not able to find financial help in the country. Shanee et al. (2015) report that the mean costs of bureaucratic procedures to set up a PPA in Peru is USD 22,000. Because of the significant start-up costs and lack of certainty, there is concern that permanent easements or similar long-term agreements may demand a level of commitment that will deter other landowners (Freese et al. 2010). Kamal et al. (2015b) also detail the lack of incentives for PLC in Poland. Additionally, because the success of private approaches is dependent upon the structure and financial robustness of the operating entities, large international NGOs have a competitive advantage, with more resources at their disposal than their smaller, independent counterparts on the ground, particularly in developing countries (de Santo 2012).

#### Skills and resources

Land trusts have been challenged by poor communication, lack of clarity around roles and responsibilities, and duplication of effort (Ryan et al. 2014). Some question whether private landowners have sufficient expertise for long-term conservation (Pasquini et al. 2011; Holmes 2015). Gaps highlighted specifically for easement holders included time to undertake management actions on the properties and, for NGOs, capacity for spatial mapping and dispute resolution (Lindsay 2016; Rissman et al. 2017).

#### Information

Lack of access to information about PLC can hinder research and planning activities. Systematic conservation planning requires knowledge of which lands are already protected, and the characteristics of those lands, to make the most effective decisions about future purchases (Rissman et al. 2017). A lack of coordination of and access to spatial data appears to be common to conservation land trust organizations (Ryan et al. 2014).

In addition to planning, information is needed for outcomes measurement and transparency. Lack of consistency in measurement in Australia’s covenanting programs, for example, meant that no positive program outcomes could be identified with certainty (Fitzsimons and Carr 2014). The privacy associated with conservation easements has become a barrier to aggregating US conservation easement data and making it available to the public (Morris and Rissman 2009), where the right to information and the right to privacy sometimes conflict (Rissman et al. 2017).

#### Accountability

In some contexts, there is concern that private landowners who receive government benefits lack accountability to the public, which has little access to information about the effectiveness or general benefit of government investments into PLC (Mitchell et al. 2017). Private for-profit operations may involve a lower degree of public participation, consensus orientation, accountability, transparency, and equity than occur with non-profit organizations (Eagles 2009). Approaches for reducing legal and administrative barriers to accountability include binding reporting requirements to government funding for conservation on private land, as is the case with the Farm Bill in the US (Rissman et al. 2017).

### Group 2: Value Conflict

Articles with themes in Group 2: Value Conflict generally situate their findings in the context of theory from political ecology and critical geography. Many of the issues in Group 2 are not specific to PLC, but reflect broader societal concerns, with PLC as one realized instance (of potentially many) of the factors of concern.

#### Cultural conflict

Cooke and Corbo-Perkins (2018) identified four tensions between the market logic of an Australian reverse auction program and the conservation practices of landowners. First, some landholders used the payment scheme to increase regulatory protections on their property through covenants, which appears contradictory because market-based instruments are often set in contrast to regulatory mechanisms. Second, many landholders struggled to conceive of their stewardship practice as contractual labor, although the nature of the program necessitated it. Third, due to anthropogenic changes to the ecosystem, the landscape did not respond to restoration efforts that aimed to return land to the program’s ecological benchmark goals; landholders were instead producing novel ecosystems. Fourth, many landholders wanted social collaboration with other auction participants, whereas the program required competition for cost efficiency.

In Peru, assigning market value to nature may discourage local people from conserving wildlife for its intrinsic or cultural value and promote land trafficking and corruption (Shanee et al. 2015). Yet regulation may not provide a complete solution, either. Cousins et al. (2010) document a conflict between regulation and profit and say that new regulations in the South African wildlife industry will encourage some ranchers to convert their land away from conservation-friendly land use.

PPAs owned by people from outside the proximate geographic area may find their practices conflict with traditional values of local communities and indigenous groups. Questions have long been raised about the way international NGOs in the global north operate in the global south, where they can reinforce a separation of nature and culture (de Santo 2012). In Chile forest-dependent cultures with traditional utilitarian values toward nature collide with contemporary land-protection strategies, such as restricted access, and local residents do not share beliefs about the benefits of large-scale private development-based conservation (Serenari et al. 2015). Community leaders did not see a match between community goals and the opportunities for conservation and economic development offered by the PPA. For example, a community member working as a guard for a PPA felt that his people had deserted their indigenous culture, which was based on communication and collective action, and in its place embraced values rooted in individuality, competition, self-interest, and inequity (Serenari et al. 2017).

#### Inequity

Community members living near a PPA in Chile perceived landowners as wealthy, powerful, and privileged and thought that, relative to communities, it was landowners who reaped the returns of PPA tourism (Serenari et al. 2017). Within the community, individuals who worked for the park or in tourism benefitted more than those who did not. In integrated conservation and development programs, which can include PPAs with a dedicated development component, programs may offer temporary solutions and their benefits be realized at the global rather than the local level (Serenari et al. 2015).

Inequity can also be found at the organizational level. For example, the system for private marine protected areas appears to favor large, international NGOs, which have more resources at their disposal, and disfavor small ones, especially in developing countries (de Santo 2012).

#### Neocolonialism

Historically, in South Africa, the Natives Land Act of 1913 and successive laws prohibited Africans from buying or owning land outside of designated “native reserves” (Spierenburg and Brooks 2014). Today, charges of neocolonialism are relevant where those from outside a culture import and impose ideas about conservation and development. In Chile, for example, indigenous communities appear to have adopted some of the values of colonists, leading to overexploitation of land (Serenari et al. 2017). Foreign ownership of private parks opens itself to the charge that it is a subtle form of neocolonialism; foreign ownership should therefore be approached cautiously, especially in lesser-developed countries (de Santo 2012).

#### Exclusion

PLC is premised upon private land ownership, a concept that is itself tied to colonialism in many places. In South Africa, the history of private land ownership is closely linked to the settler land dispossessions that took place in the nineteenth and early twentieth centuries (Spierenburg and Brooks 2014). Today, mechanisms of exclusion can include active expulsion of people claiming rights to land. Exclusion may occur through “securitization,” when military-style security is used to guard protected areas against poaching, enabled through private security companies, national armies, and soldiers for hire (Massé and Lunstrum 2016).

A less direct mechanism is pricing. Some reserve owners use a model of ecotourism that targets limited numbers of wealthy tourists in order to minimize the ecological impact of tourism; such lodges are unaffordable to the majority of the population in the country (Ramutsindela 2015). Related to this, the marketing of conservation as pristine wilderness devoid of human influence can fuel anti-agrarian bias (Spierenburg and Brooks 2014).

Exclusion can also be political. A case study in Paraguay found local people excluded from all governance and decision-making (Quintana and Morse 2005), and community members in Chile reported feeling disempowered by exclusion from decision-making at a nearby PPA (Serenari et al. 2016). The impact of exclusion on local people can be severe. In Indonesia, locals who lost access to land due to ecosystem restoration concessions were forced to abandon their traditional forest-based livelihoods and jobs as laborers for the forestry industry (Buergin 2016). Exclusion from subsistence and commercial use of land on PPAs has also been documented in Chile and Mozambique (Meza 2009; Massé and Lunstrum 2016; Serenari et al. 2017). Serenari et al. (2016) found that PPA administration and national government officials viewed local people as threats to forest conservation goals.

Exclusion is detrimental not only to local people, but also to conservation efforts. In Mozambique, residents evicted from protected areas have shown resistance through activities like poaching (Massé and Lunstrum 2016). Poverty and resentment toward wealthy private landowners have led to conflict between private conservancy managers and the owners of group ranches in Kenya, as the latter provide an access point for poachers to enter private conservancies (Sundaresan and Riginos 2010). There have also been occupations of private properties and occasional violent attacks on conservancy managers.

#### Self-serving motives

Some people perceive PLC to be self-serving, arguing that it is not driven by altruistic motives, but as a way to promote and protect self-interest. Ramutsindela (2015) implicates PLC in his broader critique of philanthropy, arguing that philanthropic activities reflect the interests and biases of donors and promote values of social order and economic development favorable to the powerful. He argues that private nature reserves in South Africa are used to achieve three interrelated objectives: to deter land claims, give wealth-generating activities a “human face,” and control a labor pool for purposes of upmarket ecotourism ventures. This echoes findings by Serenari et al. (2016) that PPA administrators avoid participatory democracy approaches to ensure local resistance does not threaten their authority.

Owners of PPAs have also been accused of using conservation as a marketing ploy, effectively greenwashing other activities (Barany et al. 2001; Meza 2009). In some situations, PPAs have attempted to offset negative local impacts by developing grant-making, security, and economic development programs in communities. These, too, have been the target of criticism. In South Africa, PPAs are accused of using philanthropy to “tame” land claimants, reducing the likelihood of claims (Ramutsindela 2015). Similar offers of financial security through livelihood opportunities in Chile were interpreted by communities as a bribe (Serenari et al. 2016).

In the US, conservation easements appear increasingly to serve nonconservation interests of landowners by incorporating allowances for additional developments. For example, one easement reviewed by Rissman et al. (2007) included allowances for new residential and ranching buildings, a tennis court, swimming pool, and other outbuildings, and another had half its area in a building envelope. This trend could be the result of shifting demographics among landowners seeking easements and may suggest that a growing proportion are interested in securing easements as strategic investments rather than from a predominantly charitable intent.

#### Agenda-setting and control of discourse

PLC provides a context for issues of agenda-setting and control of discourse. Morgan (2008) raises concerns over who gets to set the terms and goals of environmental initiatives and what happens to those who wish to redefine or reject agendas not of their making. Easements, for example, privatize and rescale a great deal of land conservation decision-making authority (Morris 2008). Likewise, market-based incentives limit possibilities for local communities to seek their own path to conserve nature and protect human well-being outside of global economic markets (Serenari et al. 2015).

Agenda-setting can also take place in formal policy environments. In the realm of private marine protected areas, NGOs serve as unofficial diplomats (de Santo 2012), representing the interests of their constituencies by engaging in information exchange, conducting informal negotiations, and providing policy advice in ways that affect the outcomes of multilateral environmental agreements; however, they do so with no formal standing to accomplish these objectives as surrogates for state actors.

#### Elitism

Not all PPAs limit public access, but where they do they risk becoming “islands of elites” where wealthy landowners host affluent tourists (Langholz and Krug 2004; Meza 2009). Concerns over elitism and inequity can lead to local dissent against conservation activities (Serenari et al. 2015).

#### Neoliberalism

Market-based instruments, conservation easements, philanthrocapitalism, and the rise of business markets and logics are cited as examples of the influence of neoliberalism on land conservation. PPAs that embrace market mechanisms, such as payments for ecosystem services, may enable a reduction in direct state intervention in conservation, and they can be accompanied by discourses suggesting that markets are the only way to effectively conserve biodiversity while producing social benefits (Holmes 2015). Easements are seen to exemplify neoliberal environmental governance, as they privatize conservation decision-making authority; are market based; provide financial incentives for participation rather than punish noncompliance; and commodify new property rights (Morris 2008).

Neoliberal governance is also characterized by outsourcing functions traditionally performed by government to the private sector. PPAs may be seen as outsourcing public responsibility for conservation to the private sphere. This occurs, for example, on the border of Kruger National Park in South Africa, where the central government delegates functions of state security to private reserves, enabling new actors to participate in governance activity (Massé and Lunstrum 2016). While nonstate actors have always been involved in conservation, the wider range of actors seen today are thought to be more deeply embedded in capitalist networks, operating across scales.

Criticism of neoliberal conservation assumes that conservation should confront, not concede to, neoliberalism as a governance paradigm. Holmes finds that PPAs “provide only a limited challenge to the social and environmental consequence of the integration of southern Chile’s natural resources into global neoliberal economic chains” (Holmes 2015, p. 850) and that there is no evidence that PPAs challenge the broader paradigms of natural resource use outside of their boundaries.

#### Depoliticization

In some situations, PLC may serve as a mechanism to depoliticize conservation and avoid addressing broader social justice issues. For example, in Mozambique, security interests serve as a depoliticized alibi for explicit accumulation of property through conservation-related dispossession (Massé and Lunstrum 2016). In Chile, PPA campaigners are sometimes unwilling to challenge the social and environmental consequences of national resource commodification to avoid reduced political support for their cause (Holmes 2015).

#### Commodification

Some PLC tools, such as easements, are seen to be instrumental in commodification of new property rights (Morris 2008). Other actions characterized as commodification include NGOs based in the global north using simplified and stylized imagery of nature and people in the global south to market their programs to potential funders and the public (de Santo 2012). International NGOs operating PPAs in Chile initiated capacity-building programs in local and indigenous communities that were concentrated on skill-building in a market-based economy, essentially commoditizing labor (Serenari et al. 2016). In addition, some commentators have argued that the construction of a commodified wilderness is a precondition to green grabbing (see below) (Spierenburg and Brooks 2014).

#### Economy and employment

Economic impact varies by type of protected area and location. PPAs that operate in the high-end tourism market in South Africa may generate more employment than other land uses, such as livestock ranching, but many local farm dwellers can access only low-income service jobs rather than the more lucrative jobs like wildlife guiding (Spierenburg and Brooks 2014). There appears to be a net loss of employment for PPAs financed through hunting, venison production, or the breeding of wildlife for trade, compared with the agricultural enterprises that they replace.

Even where economic impacts are positive, issues can arise when actual benefits fail to meet expectations. In Chile, some local communities expected that new parks would create employment, but were disappointed by the lack of jobs and communication (Serenari et al. 2017). Unfulfilled expectations of economic benefits promised by many NGOs and governments could potentially threaten the future of some private reserves (Shanee et al. 2015).

#### Adjacency impacts

Adjacency impacts result from a proximate physical location to PLC activities. Negative impacts noted include rising land costs, tourism traffic, damage to public infrastructure (such as roads), and pollution, including dust and air pollutants (Serenari et al. 2015, 2017).

#### Disruption of local social systems

Disruption of social systems occurs when outside entities influence economic, power, and gender dynamics in a community. Serenari et al. (2015) found local dissent over livelihood disruption and land rights around PPAs in Chile, disproportionately benefiting some members of a community over others. In some cases, local communities believed that powerful outside entities were using disruption intentionally, attempting to divide and conquer indigenous people in this area (Serenari et al. 2016).

In Costa Rica, some landowners reported joining a national PLC program to restrict local, pro-development government authorities from undermining landowners’ efforts to protect the land’s natural state, preferring the jurisdiction of national-level agencies to local politicians (Langholz et al. 2000). This led to tension not within the community, but between national and local governments.

#### Dependence

Some PPAs foster dependence in local communities. Hora (2017) found that communities around a PPA in Chile became dependent on it as the main generator of economic activity in the area. Without a diversity of jobs, young people moved away from the area after high school graduation in search of other opportunities. Also in Chile, Serenari et al. (2017) found a community in an ongoing power struggle over community self-sufficiency. Community leaders passed a resolution preventing communities from establishing formal relations with a PPA in an effort to avoid “assistencialism,” or dependence on handouts.

Ramutsindela (2015) reported that communities adjacent to PPAs in South Africa were forced to make difficult choices between either receiving philanthropic support from the PPA or filing land claims seeking reparations for previous dispossession from the land on which the PPA was located. In such cases, accepting philanthropic support from a PPA could lead to financial dependence to the point that communities might forego risky but more autonomous political solutions.

#### Green grabbing

Land grabs are transfers of control over property and resources from local control to more powerful outsiders, and green grabs are land grabs undertaken primarily for environmental reasons (Holmes 2014). In Chile, powerful actors have acquired control over land and resources over the last 150 years, often facilitated by the Chilean state. Holmes (2014) reports there is no indication that any PLC transactions were forced, compelled, or unjust, except for two questionable cases, but there is potential for PPAs to be part of the ongoing process of change in land control in Chile.

Others have expressed concerns over the foreign ownership of private reserves. Langholz and Krug (2004) pointed to research finding that a third of private reserves in Africa and a fifth in Latin America were completely owned by foreigners. Additional concerns in this area include the contribution of commodified wilderness and the securitization of conservation to the green grabbing phenomenon (Spierenburg and Brooks 2014; Massé and Lunstrum 2016).

#### Accumulation and control of land

Langholz and Krug (2004) note that private parks can contribute to the concentration of land ownership by the wealthy. Some landowners appear to enroll in conservation programs to protect land from redistribution by government (Langholz et al. 2000). PLC also includes efforts by for-profit entities (Stolton et al. 2014); in this light, Ramutsindela (2015) argues that accumulation is integral to PLC. He states that the private sector sees conservation areas as a niche market for capital accumulation, with companies in particular using PLC to develop their competitive advantage. In Mozambique, for example, the creation of a new private wildlife tourism concession resulted in over 90 local community households being dispossessed from their homes, with the possibility of more in the future (Massé and Lunstrum 2016).

#### Legitimacy

Issues of legitimacy arise where local communities and other stakeholders question the ownership or use of PPAs. In cases of land claims, such as in Chile or South Africa, private land ownership rights are challenged through the legal system (Meza 2009; Ramutsindela 2015). In other cases, such as privately-owned conservancies in Kenya, the landowners tend to be of foreign origin, are not accepted as part of the community, and thus find it harder to gain political support for their causes (Leménager et al. 2014).

### Group 3: Economic Inefficiency

Only two articles included themes found in Group 3: Economic Inefficiency. Nevertheless, they warranted their own group because they had little overlap with themes in other groups. Concepts are aligned with ideas in neoclassical economics and libertarian thought. Similar to Group 2, the concepts are not unique to PLC but relate to broader societal issues.

#### Moral hazard

In economics, the term moral hazard refers to situations in which the risks of a private activity are involuntarily shared by the wider public, thereby creating perverse incentives for private actors. Meiners and Parker (2004) argue that financial incentives, such as tax rebates, can weaken incentives for landowners to minimize the full, long-term costs of conserving environmental amenities. Tax laws encouraging perpetual easements on amenities that lack public good characteristics may also unnecessarily hinder the extinguishment of easements of marginal environmental benefit.

#### Hubris of the present

The permanent status of perpetual easements leads to concern over intergenerational equity. Meiners and Parker (2004) question why the tax code should privilege the perpetual conservation easement over alternative contracting instruments. This perspective argues that, in an environment of uncertainty, present generations should not lock in land use decisions when there is no guarantee that present day use is optimal for future generations. In Chile, a group of “defenders of the free market” were reportedly angry that landowners would purposefully and explicitly use a property for conservation purposes on a permanent basis (Tecklin and Sepulveda 2014). Their actions were attributed to a pro-market position, though they appear to be more consistent with a pro-growth position.

#### Observations

**Fig. 2:**
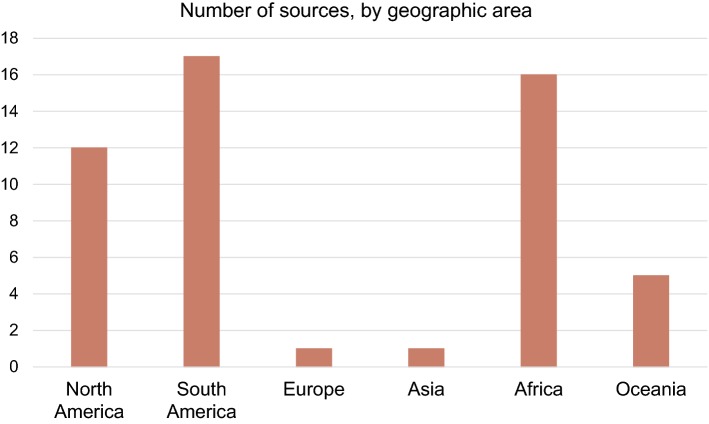
Number of source articles that focused on countries in each global region. Over half the articles focused on South Africa, the US, or Chile

**Fig. 3:**
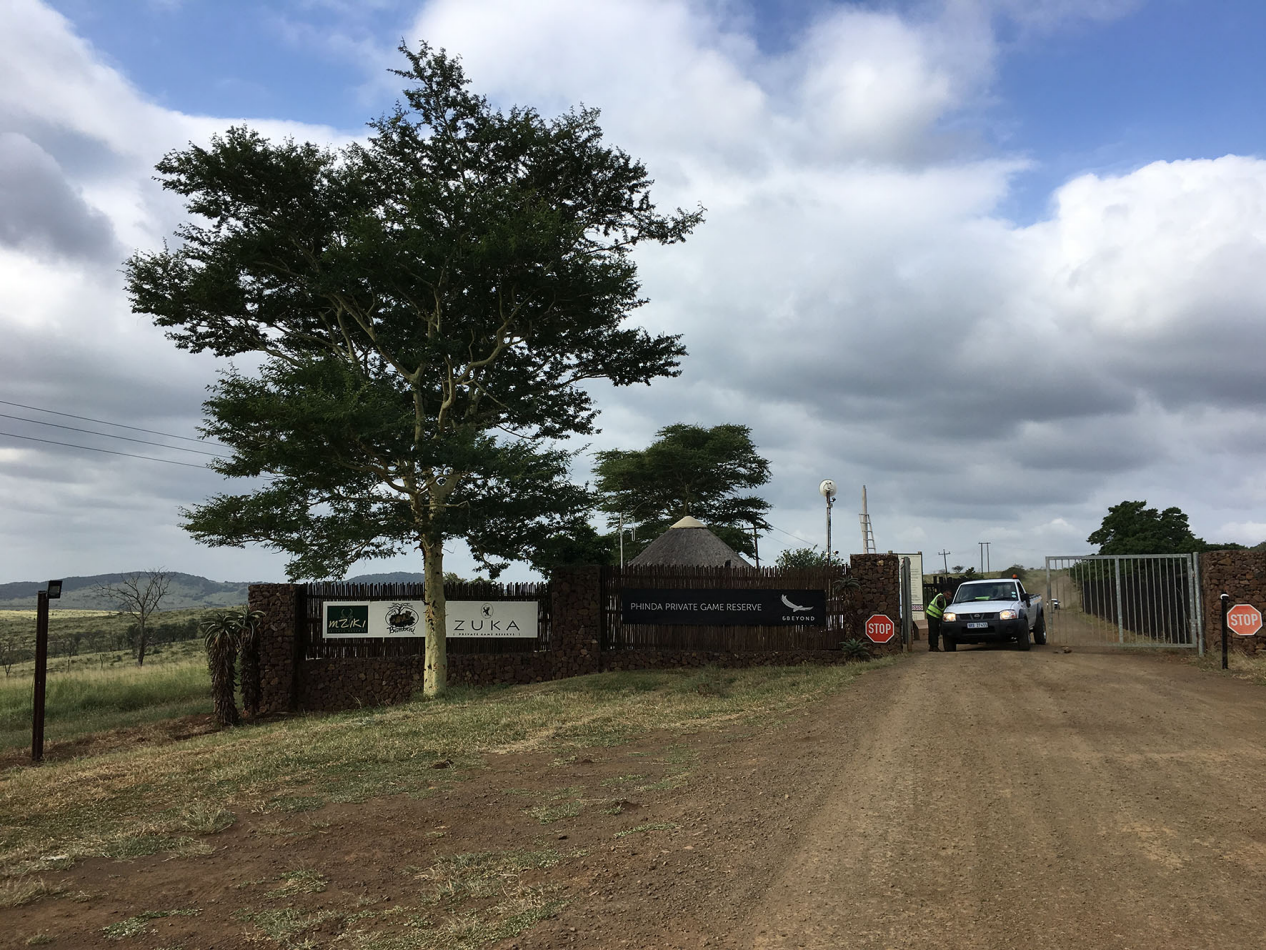
Access to some privately protected areas, such as this reserve in South Africa, is tightly controlled. Photo credit: Jennifer Gooden

Examining the overall body of critical literature from this exercise, three noteworthy issues emerge. The first, mirroring the findings of Cortés Capano et al. (2019), is evidence of a distinct geographic bias (Fig. 2). Over half the articles in our review sample focused on three countries: South Africa (Fig. 3), the US, and Chile. Although they are located on different continents, these countries have a high level of biodiversity, and they share socioeconomic systems that are premised upon strong private property rights—factors that are likely preconditions for PLC and that make the countries a target for critical analyses. However, PLC is also known to occur in many other countries. Some, such as Australia, are highly represented in the overall body of literature on PLC but less so in the critical literature, suggesting either fewer issues of concern or less interest in reporting them. Other countries, such as Brazil, have numerous PPAs but are poorly represented in the research overall, indicating a need for more internationally recognized scientific literature in underrepresented geographic regions.

The second issue is that many of the criticisms and concerns could be leveled at all types of protected areas, not merely private ones. This applies to, for example, detrimental practices associated with tourism, overemphasis on start-up funding relative to ongoing management costs, and concerns about exclusion and social disruption. The third, apparent from Fig. 1, is the closer association between effectiveness and value conflict concerns than with those of economic inefficiency, which appear relatively isolated. These three observations raise interesting questions concerning the academic discourse on PLC.

## Discussion

All conservation action takes place within contexts of complex social–ecological systems characterized by institutional arrangements that blend private, communal, and state roles in ways that vary uniquely between and across jurisdictions (Ostrom et al. 2007; Ostrom 2010). Private actors play significant roles in land conservation in certain geographic areas, but our assessment of critical views reveals that making robust generalizable observations about the global nature of PLC is challenging, due to both institutional heterogeneity and the diverse disciplinary lenses of researchers. Our thematic analysis aimed to address this issue by adopting a pragmatic, interdisciplinary, and inductive approach, allowing the emergent themes and groups to provide a landscape for further analysis.

Some patterns observed in this review may be artifacts of the search terms, which restricted the search to journal articles concerned specifically with PLC. A few articles included many themes and are therefore over-represented in the analysis, e.g., de Santo (2012), Massé and Lunstrum (2016), Serenari et al. (2015, 2016), and Spierenburg and Brooks (2014). Likewise, some relevant topics appear underrepresented relative to their importance in political ecology literature; examples include neocolonialism, inequity at both the individual and organizational levels, and elitism. These factors, however, did not compromise our primary goal, which was to catalog the topics of concern regarding PLC.

The themes identified in this literature review align with two different conservation social science approaches: research *for* conservation and research *on* conservation (Sandbrook et al. 2013). Research for conservation generally shares with conservation the normative mission to contribute to the conservation of biodiversity. In the present research, Group 1 represents this perspective. In contrast, research on conservation does not necessarily share conservation’s mission but rather studies biodiversity conservation as a social phenomenon. Here, Groups 2 and 3 represent this perspective.

The themes identified in this review can also be viewed as an issue of management versus governance. Management actions include deployment of resources, development of plans, and implementation of actions, whereas governance refers to issues of control: the structures, processes, and traditions that determine how power and responsibilities are exercised, how decisions are made, and how stakeholders have their say (Graham et al. 2003). That is, management is how conservation is done, and governance is who has the power to do it. The themes in Group 1 are primarily concerned with management effectiveness, pointing out areas where PLC activities fall short of their mission. In contrast, the themes in Group 2 and 3 are questions of governance. Concerned with power, authority, and responsibility, analytical work on these themes points to areas where PLC is in conflict with other social values. Currently, many PPAs are not well equipped to address these issues.

**Fig. 4:**
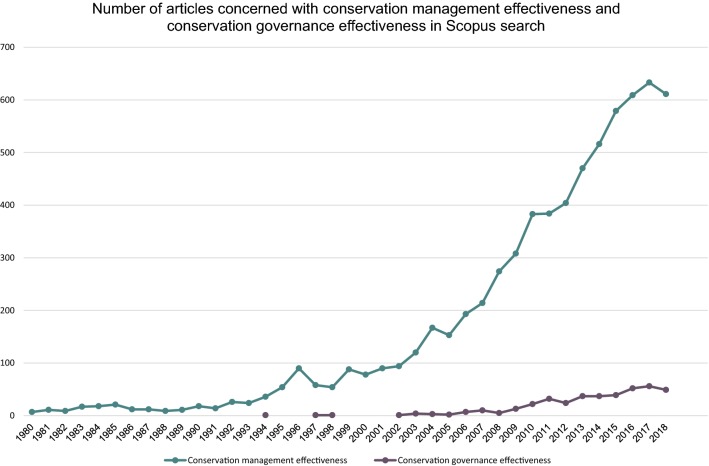
In the context of conservation, governance effectiveness is a newer and less-studied topic than management effectiveness, based on a Scopus search

Though conservationists have noted the importance of management effectiveness for decades, governance effectiveness has been slower to gain ground. For comparison, a September 2018 search in Scopus for *conservation management effectiveness* returned 6893 articles published since 1980, with an increased rate of publication since 2005. A similar search for *conservation governance effectiveness* returned only 397 articles, 90% of which have been published since 2009 (Fig. 4).

Lockwood’s (2010) framework of seven principles for assessing governance effectiveness (Table 3) can be usefully applied to PPAs. The principles can be operationalized by identifying the governance outcomes that are implied by each, using an evidence-based qualitative methodology (e.g., Lockwood 2009). Its application to PPAs could help steer them toward practices that are just, equitable, and representative, resulting in broader mainstream support.

**Table 3:** Principles and sample concerns from Lockwood’s (2010) governance effectiveness framework

Principle	Sample concerns
Legitimacy	Bestowed authority or earned legitimacy
	Actions in accordance with mandate and with integrity and commitment
Transparency	Governance and decision-making open to scrutiny
	Making evident achievements and failures and the reasoning behind decisions
Accountability	Acting within roles and responsibilities
	Downward and upward accountability
Inclusiveness	Stakeholders have opportunity to participate in processes and actions
	Outreach to marginalized stakeholders
Fairness	Interpersonal respect; decisions without bias
	Intrinsic value of nature
Connectivity	Connection across multiple levels of governance
Resilience	Culture of learning and adaptability
	Balance of flexibility and security
	Procedures to assess and manage risk

The Lockwood framework discusses stakeholder inclusion, but it does not offer guidance on the question of who counts as a stakeholder in PLC. Broadly, stakeholders are the people and organizations who affect or are affected by a decision (Sterling et al. 2017), but the implications of this definition vary by context. A Group 1 or Group 3 perspective might look at this question through the lens of property rights, concluding that, on a property obtained by market transaction or charitable donation, the owner has authority over land management decisions. Outreach to local communities might be considered either a gesture of goodwill or a pragmatic means of achieving conservation objectives. A Group 2 perspective might draw a larger circle when identifying stakeholders, emphasizing the rights of local communities, former inhabitants or land users, and other affected parties.

Regardless of landowners’ obligations, in at least some cases, inclusion of a range of stakeholders can lead to better, more viable decisions (Kerr and Tindale 2004). Pragmatic arguments for inclusion of stakeholders include: (1) a diversity of inputs in decision-making may lead to higher quality decisions that are better adapted to the local sociocultural and environmental contexts; (2) collaborative decision-making leads to the development of common ground, trust, and reduction of conflict; and (3) stakeholder buy-in may increase support and successful implementation; and (4) inclusion can lead to reduced implementation costs (Sterling et al. 2017).

Looking forward, this review points to three areas for further research. First, we have noted the distinct geographic biases in the critical literature. More research is necessary to determine if the issues that manifest in these countries are relevant in other geographic, cultural, and political environments.

Second, additional research is necessary to determine which issues in this body of literature are specific to PLC. It appears that some, such as permanence and access to information, are directly relevant to the private governance of protected areas. However, in all three groups, many issues appear to lack specificity to PLC or even to conservation. Instead, they illustrate that conservation is a social phenomenon embedded within cultures and institutions that are themselves flawed.

Finally, there remains a question of the extent to which this literature is representative of the views of the broader cultures in which PLC is embedded. The present analysis reveals a distinction between Group 1, which is not overtly political, and Groups 2 and 3, which are political perspectives. Group 2, associated with a left-leaning or progressive political perspective, is much more thoroughly developed than Group 3, which is associated with a right-leaning or conservative perspective. This is reflective of the critical landscape of academia, but its reflection of a broader population is unclear and needs further attention.

If in fact we can extrapolate from critical academic analysis to the concerns of broader society, this review implies that addressing governance effectiveness would enable PLC advocates to alleviate some concerns about PLC. A challenge here will be balancing the conflicting goals of landowner autonomy and broader accountability. For landowners, autonomy is part of the appeal of owning privately protected areas (Gooden and Grenyer 2019). Governance principles such as inclusiveness, which emphasizes participation of all stakeholders, and connectivity, which emphasizes connection across levels of governance, may pose particular difficulties. However, if PLC advocates can engage landowners in discussion, there is an opportunity to improve the way in which conservation is conducted. The present review highlights a range of issues that can be added to the discussion agenda.
